# Age‐Related Differences in Response Time Across Adolescence Reflect Premotor, but Not Motor, Processing Speed

**DOI:** 10.1111/psyp.70313

**Published:** 2026-05-06

**Authors:** William Slawson, Greg Hajcak, Bob McMurray, Bruce D. Bartholow

**Affiliations:** ^1^ Department of Psychological and Brain Sciences University of Iowa Iowa City Iowa USA; ^2^ School of Education and Counseling Psychology Santa Clara University Santa Clara California USA

## Abstract

Extant literature suggests that developmental improvements in processing speed reflect changes in a common global processing factor. In theory, then, the influence of age on processing speed should be shared across premotor processes (e.g., response selection) and motor processes (e.g., response execution). However, some researchers have observed differences in the effect of age on speed across different processes depending on stage of development, and research on neurodevelopment has long demonstrated variation in the developmental trajectory of cortical regions associated with different functions. The current study explored whether age‐related differences in processing speed during adolescence varied between premotor and motor domains, testing whether these domain‐specific differences accounted for age‐related variance in choice reaction time (RT). Adolescent participants (*N* = 204, 68.6% female) varying in age from 14 to 19 years (*M*_age_ = 16, SD_age_ = 1.73) completed a flanker task while EEG was recorded. We quantified the lateralized readiness potential (LRP) to fractionate RTs into premotor (stimulus‐locked LRP [S‐LRP]) and motor (response‐locked LRP [R‐LRP]) intervals. Both S‐LRP and R‐LRP latencies correlated with RT, but only S‐LRP latency decreased with age. Mediation analysis confirmed a significant indirect effect of age on RT through S‐LRP latency but not R‐LRP latency, suggesting that faster processing speed among older adolescents stems from faster premotor—but not motor—processing. We demonstrate the utility of using LRP latencies to investigate domain‐specific processing speed, highlighting directions for future work to link structural development research to functional measurements.

## Impact Statement

This study uses a novel ERP approach to challenge the prevailing theory that all components of processing speed develop uniformly across adolescence, showing distinct age effects on premotor and motor speeds in a cross‐sectional design. Utilizing LRP onset latency to quantify speeds of distinct processes within a single task, the study demonstrates the utility of applying a psychophysiological method to studying mental chronometry in developmental contexts, bridging functional and structural accounts of adolescent brain development.

**FIGURE 1:**
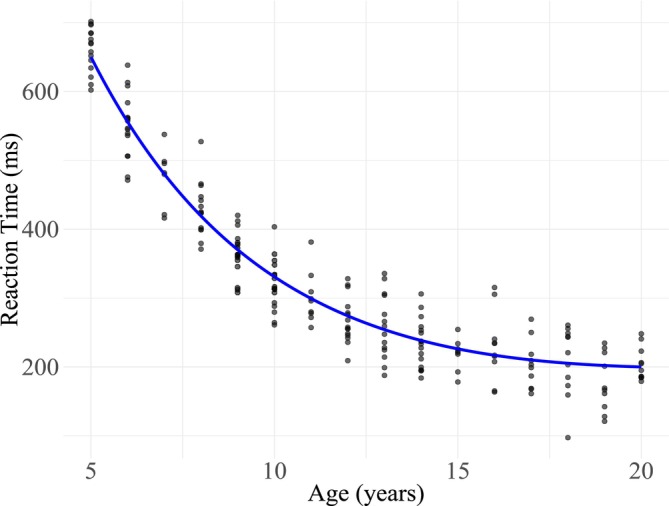
Common patterns of RT changes throughout premature development. The figure above was produced using simulated data, with reaction time used as a behavioral index of processing speed (see Kail 1991b).

Processing speed is a crucial factor in cognitive and reasoning abilities (Coyle et al. 2011; Fry and Hale 2000; Kail et al. 2016; Kail and Salthouse 1994; Nettelbeck and Burns 2010; Sheppard and Vernon 2008). The maturation of processing speed, as indexed by age‐related decreases in choice reaction time (RT), follows an exponential course (see Figure 1): increasing rapidly in early childhood, then more slowly during adolescence, before peaking in young adulthood (Salthouse and Kail 1983) and slowly declining with senescence (Birren 1965; Cerella 1985; Salthouse 2000; Salthouse and Somberg 1982; Von Krause et al. 2022).

Information‐processing frameworks of development conceptualize age‐related changes as gradual, continuous improvements in the efficiency and coordination of neurobehavioral processes (Kail and Salthouse 1994). Within this framework, age‐related differences in processing speed may reflect either the influence of single global processing factor or the cumulative maturation of domain‐specific subprocesses. Many theorists argue for the influence of a global neurobehavioral processing factor (e.g., Cerella and Hale 1994; Kail 1986, 1991a; Kail and Park 1992; Miller and Vernon 1997). Indeed, age‐related improvements in RT have been observed throughout pre‐adult development across a wide variety of laboratory tasks, suggesting improvements are driven by a single, non‐specific processing factor. Kail and Salthouse (1994) posited that this global processing factor functions within cognition like a computer's CPU—a singular component serving as a foundational parameter for speeded performance across the wider neurobehavioral system. In a meta‐analysis of the literature, Kail (1991a) fit growth curves to the patterns of RT changes across development and found that a single parameter (decay, or how fast RT drops with age) was constant across tasks, suggesting improvements can be attributed to a single factor (also see Fry and Hale 1996; Kail 1991b; Kail and Park 1992; Miller and Vernon 1997).

Yet, domain‐specific differences in how age impacts processing speed also have been observed. Kail and Miller (2006)'s longitudinal study showed that RT on language‐related tasks was faster than on non‐language tasks at age 9 but not at age 14, suggesting asynchrony in the development of processing speed across these domains. Cerella (1985) and Lima et al. (1991) found similar domain asynchrony in age‐related slowing during senescence. Taken together, this evidence suggests that a global developmental factor drives age‐related changes in processing speed, but its effects may vary across domains depending on age.

Most of the pioneering literature investigating the development of global and domain‐specific processing speed compared RTs across different tasks (Hale 1990; Kail 1991a, 1991b; Kail and Miller 2006; Miller and Vernon 1997). However, RT—the time elapsed between stimulus onset and the participant's behavioral response—comprises a combination of cognitive/premotor and motor processes (see McCarthy and Donchin 1981). Thus, simply demonstrating differences across tasks does not account for the contributions of different processing domains on RT within tasks. Continuous flow models of information processing (see Eriksen and Schultz 1979) posit that response output represents the contributions of numerous perceptual, cognitive, and motor processes, and that the speed at which these underlying processes operate is not constant across domains. Moreover, the processes themselves do not operate in a discrete, stage‐like manner; rather, the speed of one process can “leak” onto another process within the cascade (Coles et al. 1985). Arguably, then, parsing the nuances of domain‐specific development in processing speed requires a lower unit of analysis.

Evidence of asynchrony among domains contributing to the development of processing speed would be notable because it could provide insight into a potential mechanism for the nonlinear pattern of changes observed in prior research (Kail 1991b). Examining motor speed provides an opportunity for comparing age‐related speed differences among distinct processes underlying RT. Like other domains, motor speed increases rapidly in early development (Carlier et al. 1993; Denckla 1973, 1974; Gabbard and Hart 1993), with larger improvements at younger ages. Unlike other domains, however, motor speed improvements level off much earlier—usually in early adolescence (Gasser et al. 2010). This developmental trajectory aligns with neuroimaging data, which show that structural maturation in core motor and sensory areas occurs earlier than in frontal and temporal regions (Giorgio et al. 2010; Lebel et al. 2008; O'Muircheartaigh et al. 2014; Sowell et al. 1999; Tamnes et al. 2010). Yet, it remains to be determined whether this *physiological* asynchrony in the development of premotor and motor domains is reflected by similar asynchrony in their *functional* development.

Addressing this question requires a mental chronometric approach in which the contributions of distinct neurobehavioral processes to RT within a single task can be estimated (see Coles et al. 1995). To date, the most robust study of this kind was reported by Śmigasiewicz et al. (2021), who utilized electromyographic (EMG) onset of the thumb muscle to fractionate trial‐level RT into premotor time (PMT) and motor time (MT) in children aged 6–14. The authors found that PMT and MT follow the same exponential trajectory during this period, with a similar magnitude to that of overall RT, supporting a unitary process model. However, defining MT only in terms of the interval between EMG onset at the hand and full execution of a finger response obscures the role of cortical processes in motor execution. Furthermore, distal EMG onset only occurs after cortico‐spinal conduction of the motor command has reached the relevant effector muscle. Central conduction time functionally relates to improvements in motor performance (Fietzek et al. 2000; Paus et al. 1999) and follows a similar (but earlier) developmental timeline (Fietzek et al. 2000). Thus, classifying all processes prior to activation of the effector as ‘premotor’ leaves the interpretation of these findings ambiguous.

Event Related Potentials (ERPs)—especially the lateralized readiness potential (LRP)—offer a means of fractionating RT that arguably better distinguishes premotor and motor processes. The LRP is thought to reflect the processes involved in motor programming following response selection (Masaki et al. 2004), onsetting prior to cortico‐spinal conduction. Like the Bereitschaftspotential (BP), which indexes the activation of a motor response (see Jahanshahi and Hallett 2003), the LRP is a difference waveform indexing activation of the hemisphere of motor cortex initiating a response, relative to the contralateral side (Eimer and Coles 2003). Unlike the BP, the LRP reflects *preferential* activation of the chosen response channel (Coles et al. 1988). In a bivalent response task, the amplitude of the LRP indicates the level of preferential activation for the motor program of the given response, while the latency of LRP onset indicates the time at which response selection is made (see Smulders and Miller 2012).

More notable for our purposes is the way LRP latency splits RT. The LRP can be time‐locked to the onset of the stimulus (S‐LRP) or the response (R‐LRP), giving way to two distinct interpretations of onset latency. The onset latency of the S‐LRP represents the time between the stimulus onset and the initial, preferential engagement of the response channel—indexing the speed of premotor processes like stimulus evaluation and response selection that unfold prior to response activation (Smulders and Miller 2012). This inference is supported by research showing that task manipulations affecting premotor processes (i.e., stimulus redundancy, stimulus‐distractor compatibility) alter S‐LRP latency but not R‐LRP latency (Gratton et al. 1988; Mordkoff et al. 1996; Osman and Moore 1993). In contrast, R‐LRP latency represents the time between response channel activation and the overt response—reflecting the speed of motor processes following response selection. Variations in response complexity affect R‐LRP latency but not S‐LRP latency (Smulders and Miller 2012), and task manipulations aimed at biasing motor preparation (i.e., pre‐cuing) also alter R‐LRP latency (Leuthold et al. 1996; Osman et al. 1995). Based on these findings, quantifying both S‐LRP and R‐LRP latencies within a single task permits estimation of the extent to which RT independently reflects the duration of premotor (S‐LRP) and motor output (R‐LRP) processes.

LRP latencies also provide a means to examine developmental asynchrony among premotor and motor processes. The LRP is generated, at least in part, in the primary motor cortex (Coles 1989; Miller and Hackley 1992), and neural networks associated with primary motor and sensory processing reach maturity as early as 5–8 years (de Brie et al. 2012). Meanwhile, stimulus evaluation and premotor response selection are thought to be mediated by a prefrontal network (Goghari and MacDonald III 2009) for which maturation persists into early adulthood (Arain et al. 2013; Fuster 2002; Gogtay et al. 2004; Sowell et al. 1999; Spear 2000).

Only a few studies have used LRP measures to investigate immature age differences in processing speed. Ridderinkhof and Van Der Molen (1997) measured P3 latencies and S‐LRP latencies in children aged 5–12 to compare the developmental timelines of speed in stimulus evaluation and response selection processes, respectively. They showed that P3 latency (i.e., the time between stimulus onset and stimulus evaluation) decreased at a faster rate than S‐LRP latency (time between stimulus onset and response selection), with both decreasing at a faster rate than overall RT (the complete interval from stimulus onset to response execution). Considering each of these latency indicators measures the speed of an increasingly inclusive set of processes (P3 = stimulus evaluation, S‐LRP = stimulus evaluation + response selection, RT = stimulus evaluation + response selection + motor response execution), their distinct age‐related change rates suggest distinct age‐related differences across neurobehavioral processes. While P3 latency is unaffected by response selection processes (McCarthy and Donchin 1981), the temporal overlap between the P3 and LRP onset makes their relative contributions to overall RT difficult to parse. Here, we compared S‐LRP latency with R‐LRP latency because they cover distinct portions of the RT interval, thereby allowing us to compare their contributions to age‐related differences in processing speed. Szűcs et al. (2009) found prolonged S‐LRP latencies in children ages 5–8 relative to in adults, but this difference only minimally contributed to age‐related differences in RT. Similarly, Wild‐Wall et al. (2008) found that elderly adults had slower S‐LRP and R‐LRP latencies—but larger amplitudes—than young adults during a flanker task, which they suggested reflects stronger target‐based response selection in the older adults.

None of these prior studies asked whether premotor and motor processes differentially contribute to RT performance across adolescence. Most of the previous work has focused on earlier developmental periods—typically prior to age 14—when speed differences are steepest and multiple processes are maturing rapidly. In contrast, adolescence provides an informative window for testing domain‐specific patterns of age‐related differences, as some neurobehavioral processes may approach maturity earlier than others during this period. Here, we tested whether improvements in choice RT during adolescence could be differentially explained by improvements in the premotor and motor domains. Participants completed an arrowhead flanker task, from which we quantified S‐LRP and R‐LRP latencies and tested whether either LRP variable mediated the relationship between age and RT. Our primary aim was to evaluate whether age‐related reductions in processing speed (RT) were transmitted through the speed of premotor (S‐LRP) or motor processes (R‐LRP), or both. To build toward this mediation model, we first predicted that RT would decrease with age (H1), and that S‐LRP and R‐LRP latencies would make independent contributions to RT (H2), reflecting the distinct influences of premotor and motor processes, respectively. Assuming support for these hypotheses, we then asked whether age‐related changes in RT are mediated by age‐related reductions in S‐LRP latency, R‐LRP latency, or both (H3). Mediation of age‐related RT reductions by one or both LRP latencies would suggest domain‐distinct developmental processes underlying age‐related increases in processing speed, while a lack of distinct mediation through either path would suggest that RT changes instead stem from a global processing speed factor.

## 1 Method

### 1.1 Participants

Participants were recruited for a longitudinal study aimed at examining risk and protective factors for alcohol use and consequences among adolescents and young adults. The present report is based on a subsample of 204 individuals (*n* = 140 females, 64 males) who had completed the first lab visit of the parent study by 01/31/2025. Participants were drawn from two communities in Iowa (Iowa City, a small college town, and Cedar Rapids, a larger and more demographically diverse city).

Recruitment methods included: (i) mass emails sent to University of Iowa students, staff, and faculty; (ii) posted flyers; (iii) ads in local periodicals, on local radio stations, and on social media; (iv) presentations at area high schools; (v) tabling at community events; and (vi) word of mouth. Inclusion criteria for the parent study included: (1) being between 14 and 19 years old at the time of consent, and (2) being deemed at moderate or greater risk of developing problematic alcohol involvement based on age‐graded criteria developed by the National Institute on Alcohol Abuse and Alcoholism (National Institute on Alcohol Abuse and Alcoholism 2015). Exclusion criteria included (1) any history of neurological disease or disorder, (2) history of traumatic head injuries, or (3) EEG disqualifications due to high skin sensitivity or incompatible hairstyles.

Data from 10 participants were unusable due to excessive noise in their EEG (*n* = 9) or below‐chance (< 0.5) accuracy in the flanker task (*n* = 1), a standard criterion used to identify disengaged responding in cognitive tasks (Friedman et al. 2008, 2016). Statistical evaluation of chance performance using a binomial model indicated that all retained participants in the present sample exceeded the binomial threshold for above‐chance performance at α = 0.01 (approximately 57% accuracy across 300 trials).

The final sample used for analyses consisted of 194 participants across ages 14 (*n* = 30; 21 females, 9 males), 15 (*n* = 43; 32 females, 11 males), 16 (*n* = 33; 21 females, 12 males), 17 (*n* = 32; 17 females, 15 males), 18 (*n* = 33; 26 females, 7 males), and 19 (*n* = 33; 23 females, 10 males). Participants were paid $90 (USD) for completion of the lab session from which the present data were drawn.

### 1.2 Flanker Task

Participants completed an arrowhead version of the flanker task (Ridderinkhof et al. 2002) administered using EPrime Version 3.0 (Psychology Software Tools, Pittsburgh, PA) while EEG was recorded. On each trial, participants were presented with an array of five arrows in the center of the monitor and were instructed to indicate the direction of the central arrow (left or right) as quickly as possible by pressing one of two buttons on a ms‐accurate response box (Chronos; Psychology Software Tools, Pittsburgh, PA). Arrays requiring right‐ and left‐hand responses were equiprobable, as were compatible (> > > > > or < < < < <) and incompatible (< < > < < or > > < > >) arrays. Stimulus arrays were presented for 250 ms and the inter‐trial interval (ITI) varied randomly between 1080, 1100, and 1130 ms. Responses were recorded only if they occurred within 1000 ms of the stimulus onset, ensuring that all retained responses preceded the onset of the next stimulus. The task began with a 10‐trial practice block in which participants received feedback regarding response speed and accuracy. After verifying with an experimenter that they understood the task, participants completed 300 critical trials presented in three blocks of 100 trials each.

### 1.3 EEG Data Acquisition and Reduction

Electrophysiological data were captured using 32 Ag/AgCl active electrodes (BrainProducts actiCAP) positioned according to the standard 10–10 system. All impedances were kept below 10 kΩ throughout recording. VEOG and HEOG were recorded with electrodes positioned 1 cm above and below the left eye and 1 cm external to the outer canthus of the two eyes, respectively. Data were digitized at 500 Hz using an actiCHAMP Plus amplifier (Brain Products GmbH, Gilching, Germany), referenced online to the central midline electrode, and captured utilizing BrainVision Recorder software (Vers. 1.25.0204, BrainProducts GmbH, Gilching, Germany).

Preprocessing of EEG data was accomplished in BrainVision Analyzer (Version 2.3.0, Brain Products GmbH, Gilching, Germany). Data were re‐referenced offline to an average of the two mastoids and passed through a second‐order, zero‐phase shift Butterworth bandpass filter (0.1 to 30 Hz). An additional notch filter was applied at 60 Hz to minimize line noise from overhead lighting. Data were segmented separately for stimulus‐locked (S‐LRP) and response‐locked (R‐LRP) LRPs. Stimulus‐locked waveforms were segmented at −200 to 600 ms following stimulus onset, baseline corrected from −200 to 0 ms. Response‐locked waveforms were segmented at −1000 to 200 ms following button press responses and baseline corrected from −1000 to −800 ms to ensure the baseline period preceded stimulus onset, minimizing contamination from stimulus processing. All incorrect response trials were removed prior to segmentation. Ocular artifacts in all segments were corrected using the method developed by Gratton et al. (1983). A semi‐automatic artifact rejection procedure was applied to corrected segments, which were flagged for rejection if they met any of the following criteria: (1) absolute voltage exceeding ±100 μV at any time point, (2) peak‐to‐peak voltage changing ±175 μV in a moving 100‐ms interval, (3) single voltage step > 30 μV/ms, or (4) low activity signal change < 0.5 μV in a moving 100‐ms interval. Segments containing artifacts were removed (avg. 4.1% of trials per participant) prior to creating averages per electrode, condition, and participant.

### 1.4 Deriving and Scoring LRPs

**FIGURE 2:**
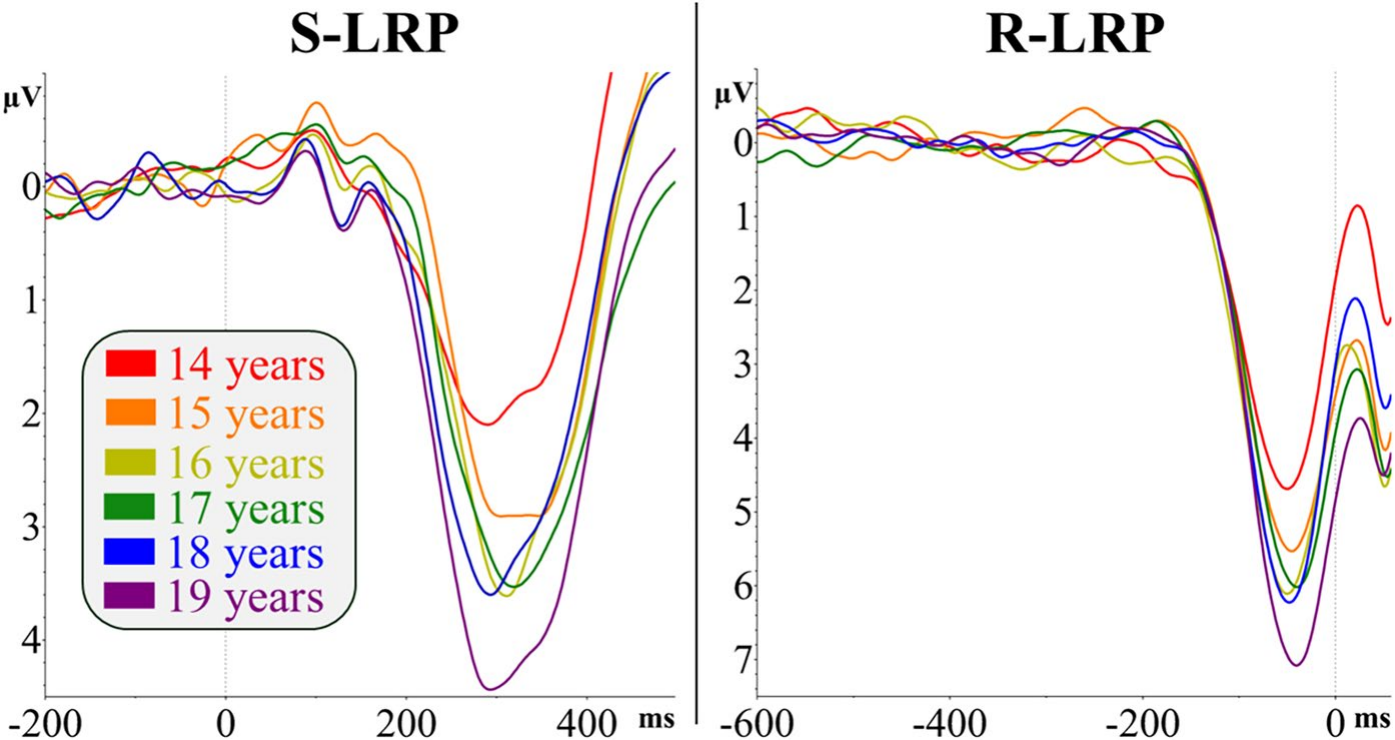
Waveforms for stimulus‐locked (left) and response‐locked (right) LRPs as a function of age cohorts. Although study analyses treat age as a continuous variable, LRPs are grand averaged in discrete age groups to facilitate visualization of age‐related differences. Grand averages were derived from correct trials. For S‐LRP, time 0 = stimulus onset; for R‐LRP, time 0 = onset of the overt response (button press). Analyses examining LRP amplitude are presented in the Supporting Information.

Figure 2 depicts S‐LRP and R‐LRP waveforms as a function of participant age. LRP waveforms were derived using the double subtraction method (de Jong et al. 1988; Eimer 1998). For all trials, activity over the right motor cortex (channel C4) was subtracted from activity over the left motor cortex (channel C3). These C3‐C4 difference waveforms were averaged separately for right‐hand and left‐hand response trials. Then, the averaged C3‐C4 difference waveform for right‐hand trials was subtracted from the C3‐C4 difference waveform for left‐hand trials, constructing an overall subject LRP.

$$\mathrm{LRP}={\left(C3–C4\right)}_{\text{left hand}}–{\left(C3–C4\right)}_{\text{right hand}}$$

LRP latencies were calculated using a version of the regression‐based method first implemented by Schwarzenau et al. (1998). Per this method, the LRP epoch is segmented with two least‐squares regression lines—one fit to the pre‐onset segment and one that rises to the peak—with the intersection of the two lines estimated to be the onset latency of the LRP. For the present analyses, we opted to use a single‐subject, 1‐df iteration of the method in which the slope of the pre‐onset line is forced to be zero, thus allowing only the intersection of the lines to vary (see Mordkoff and Gianaros 2000).

Peak latencies were detected within a window derived from the grand averaged waveforms (see Figure 2). S‐LRP peaks were detected within 200–500 ms following stimulus onset and R‐LRP peaks were detected within −200–100 ms relative to the response. Onset latency was then estimated using the regression‐based procedure within the interval spanning from the baseline period to the identified peak for each participant.

### 1.5 Analytic Approach

Before computing mean RTs for each participant, all error trials were discarded (avg. 12.0% of trials per participant), as were trials in which RTs were exceedingly fast (< 100 ms) or slow (≥ 3 SD from each participant's mean) (1.0% of trials), consistent with common RT trimming procedures (Friedman et al. 2016; Wilcox and Keselman 2003). After cleaning, participants retained M = 261 trials (SD = 21.2, range = 174–296). For both LRP latencies and overall RTs, outlying values (≥ 3 SD from the sample mean) were winsorized to the furthest non‐outlying value (0.3% of data). Age was treated as a continuous variable. To account for developmental sex differences, sex was included as a covariate in all models. All statistical analyses were performed using R (Version 2024.12.1.563). Linear mixed‐effects models were estimated using the lmerTest package (v3.1–3; Kuznetsova et al. 2017), commonality analyses were conducted using the yhat package (v2.0–5; Nimon et al. 2025), structural equation modeling was conducted using the lavaan package (v0.6–18; Rosseel 2012), and power analyses were conducted using the simsem package (v0.5–17; Pornprasertmanit et al. 2025).

Prior to hypothesis testing, we ran preliminary exploratory analyses to characterize the flanker compatibility effect and to inform model specification by testing whether the compatibility effect varied with age. This potential Age × Compatibility interaction was investigated using a linear mixed‐effects model (LME), with age (standardized) defined as a continuous between‐subjects predictor, sex as a categorical between‐subjects predictor, and trial compatibility as a within‐subjects predictor. Compatibility was coded +0.5 for compatibile trials and −0.5 for incompatible trials, while sex was coded 0 for males and 1 for females and then centered. A linear model was chosen because the sample comprised only adolescents and young adults, constituting the part of the expected exponential developmental timeline when the relationship can be best described as linear.^1^

To describe associations among key study variables, we evaluated bivariate correlations using Pearson's correlation coefficients, with significance evaluated using the associated *t*‐tests. Consistent with contemporary approaches to mediation, these associations were not used as prerequisites for testing mediation, as indirect effects can be present even in the absence of significant total or bivariate effects (Hayes 2017; Montoya and Hayes 2017). Nevertheless, in accordance with our hypotheses, we determined a mediation test to be supported by the presence of associations corresponding to each of the predicted paths: (1) age and RT, (2) both LRP latencies and RT, and (3) age and one or both LRP latencies.

To justify separate paths for each LRP latency in the mediation model, we evaluated the independence of S‐ and R‐LRP latencies in accounting for age and RT variance using commonality regression (see McMurray et al. 2025, for a tutorial), a technique consisting of a series of hierarchical regressions of shifting order which decomposes the total *R* ^2^ into the unique and shared contributions of predictors to the dependent variable.

Study hypotheses were finally tested using a structural equation model (SEM) applied with the lavaan package in R. The model tested whether age predicted RT indirectly via its influence on S‐LRP and R‐LRP latencies. Specifically, separate a‐paths were specified from age to S‐LRP latency and R‐LRP latency, and separate b‐paths were specified from each latency to RT. A direct c‐path from age to RT was also estimated, and sex was included in the model as an independent influence on RT. Indirect effects were computed as the product of the corresponding a‐ and b‐path coefficients, and the total effect of age on RT was computed as the sum of the direct and indirect effects (Montoya and Hayes 2017). Absolute model fit was evaluated based on chi‐square, Root Mean Square Error of Approximation (RMSEA), and Standardized Mean Square Residual (SRMR), while relative model fit was evaluated based on the Comparative Fit Index (CFI) and the Tucker‐Lewis Index (TLI). Consistent with recommendations in the SEM literature (Hu and Bentler 1999), RMSEA values ≤ 0.06–0.08, SRMR values ≤ 0.08, and CFI and TLI values ≥ 0.90–0.95 were interpreted as acceptable fit. Because the purpose of the present model was to test indirect pathways between age and RT rather than reproduction of the full covariance structure among variables, global fit indices were reported descriptively rather than as strict criteria for model adequacy. S‐LRP and R‐LRP latencies were mean‐centered to improve interpretability (Schielzeth 2010), and standardized coefficients were reported. Achieved power for mediation paths was estimated using Monte Carlo simulation (5000 replications) based on the fitted SEM parameters.

Exploratory analyses assessing interactions between S‐LRP latency, R‐LRP latency, and sex, as well as the relationship between age and LRP amplitude can be found in the Supporting Information.

## 2 Results

### 2.1 Preliminary Examination of the Flanker Effect

**FIGURE 3:**
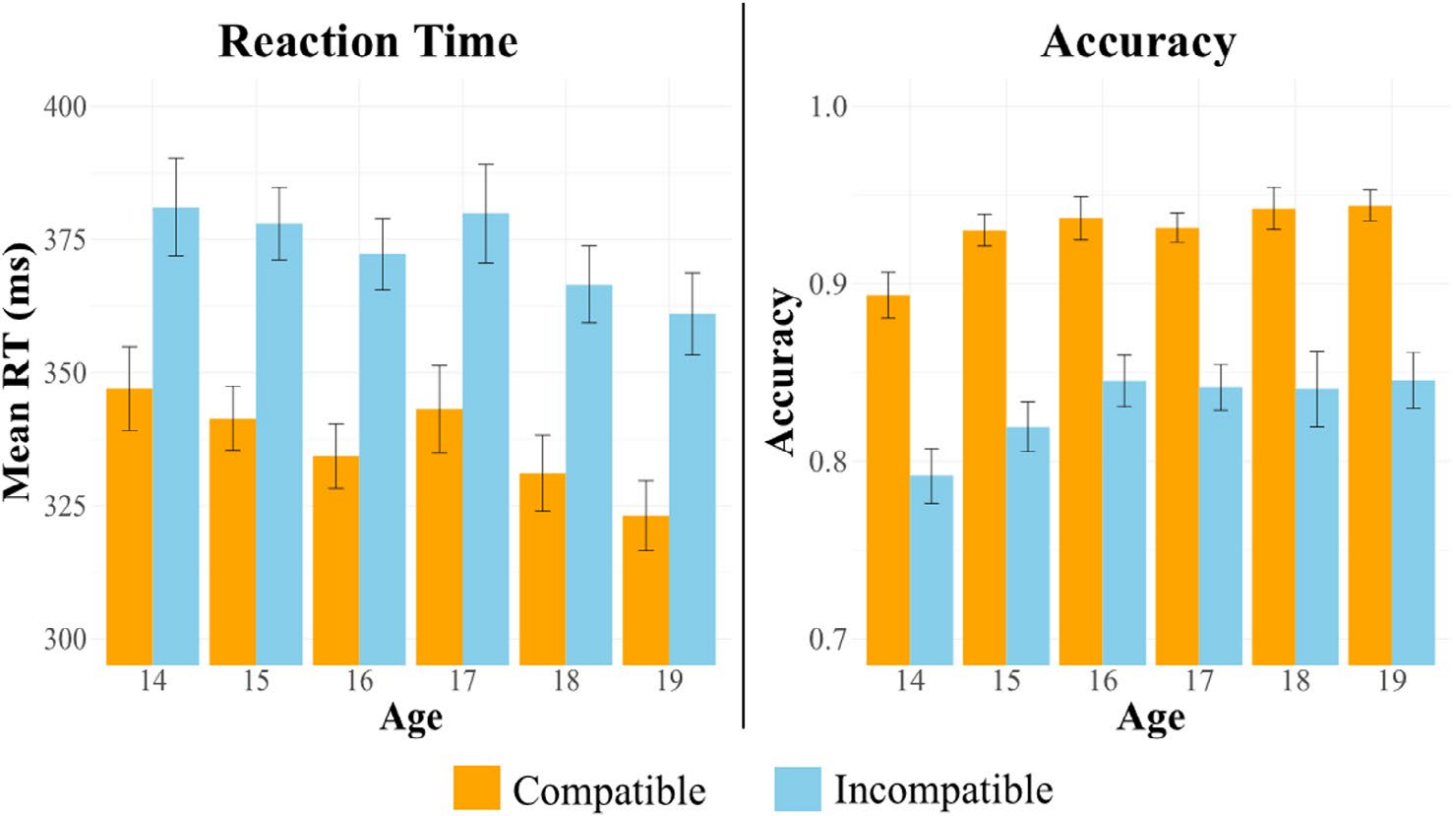
Mean RT and accuracy as a function of age and flanker compatibility. Error bars represent ±1 SE. Accuracy is presented as proportion of correct responses.

Mean flanker RT and accuracy as a function of age and flanker compatibility are presented in Figure 3. The flanker compatibility effect was characterized using an LME with age, compatibility, and sex as fixed effects with all interactions specified, and random intercepts and random slopes for compatibility by participant. Separate models were run to characterize the flanker effect on both RT and accuracy.

In the RT model, the fixed effects explained 6.9% of the variance (marginal *R* ^2^ = 0.069), while the full model with random effects explained 30.4% of the variance (conditional *R* ^2^ = 0.304). The analysis showed a large main effect of compatibility (*β* = −36.51, *t* = −34.77, *p* < 0.001), confirming the typical flanker compatibility effect, and a main effect of sex (*β* = 21.64, *t* = 21.64, *p* = 0.001), indicating that males responded more quickly than females.

In the accuracy model, fixed effects explained 2.8% of the variance (marginal *R* ^2^ = 0.074) and the full model with random effects explained 7.4% of the variance (conditional *R* ^2^ = 0.028). The model also showed main effects of age (*β* = 0.01, *t* = 2.97, *p* = 0.003), sex (*β* = 0.03, *t* = 2.73, *p* = 0.007), and compatibility (*β* = 0.10, *t* = 20.78, *p* < 0.0001). Older participants were more accurate than younger ones, females were more accurate than males, and accuracy was higher on compatible relative to incompatible trials. None of the two‐ or three‐way interactions between age, compatibility, and sex were significant (for full output, see Table 1).

**TABLE 1:** Linear mixed‐effects model results for behavioral data.

Predictors	RT				Accuracy			
	*β*	SE	*t*	*p*	*β*	SE	*t*	*p*
Age	**−6.077**	**2.892**	**−2.101**	**0.037**	**0.014**	**0.005**	**2.966**	**0.003**
Compatibility	**−36.511**	**1.050**	**−34.774**	**< 0.001**	**0.099**	**0.005**	**20.775**	**< 0.001**
Sex	**21.644**	**6.237**	**3.470**	**0.001**	**0.028**	**0.010**	**2.725**	**0.007**
Age × compatibility	−0.573	1.055	−0.543	0.588	−0.002	0.005	−0.505	0.614
Age × sex	−5.626	6.414	−0.877	0.381	0.011	0.011	1.074	0.284
Compatibility × sex	−2.371	2.278	−1.041	0.299	−0.017	0.010	−1.652	0.100
Age × compatibility × sex	2.050	2.350	0.872	0.384	−0.014	0.011	−1.274	0.204

*Note:* Significant (*p* < 0.05) predictors are bolded.

Given the absence of age differences in the compatibility effect in behavior, compatibility condition was not included as a factor in subsequent analyses. A test for invariance across trial types pertaining to the primary SEM is included in the Supporting Information.

### 2.2 Descriptive Associations Between Study Variables

**FIGURE 4:**
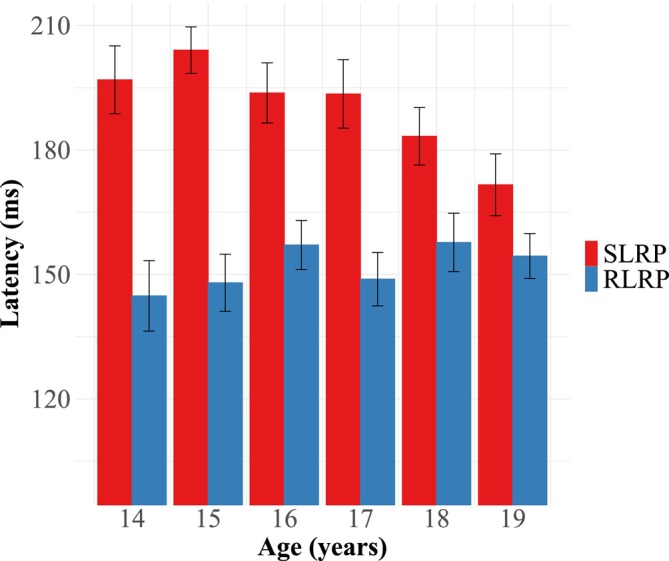
Changes in S‐ and R‐LRP latencies by age. Error bars represent ±1 SE.

Table 2 presents zero‐order correlations between age, accuracy, RT, and LRP latencies. Age correlated modestly with RT (*r* = 0.16, *p* = 0.031). Both S‐LRP latency (*r* = 0.40, *p* < 0.0001) and R‐LRP latency (*r* = 0.18, *p* = 0.014) positively correlated with RT, though the magnitude of the correlation was larger for S‐LRP latency than for R‐LRP latency (Fisher's r‐to‐z test: *z* = 2.116, *p* = 0.027). S‐LRP latency negatively correlated with age (*r* = −0.23, *p* = 0.002), while R‐LRP latency did not (*r* = 0.09, *p* = 0.229) (see Figure 4). A second Fisher's r‐to‐z test again confirmed that the correlation between S‐LRP and age was significantly stronger than the correlation between R‐LRP and age (*z* = −2.90, *p* = 0.004). Together, the observed correlations supported testing our hypotheses using a mediation model.

**TABLE 2:** Descriptive statistics and correlation matrix for all variables in our primary analysis.

	M	SD	Sex (F)	RT	ACC	S‐LRP	R‐LRP
Age	16	1.7	−0.03	**−0.16**	0.**21**	**−0.23**	0.09
Sex (F)	—	—		0.**24**	0.**19**	0.04	0.02
RT	353.70	41.76			0.**22**	0.**40**	0.**18**
ACC	0.88	0.07				0.01	0.09
S‐LRP	191.05	42.28					0.14^a^
R‐LRP	151.73	38.26					

*Note:* Significant (*p* < 0.05) correlations are bolded.

Abbreviations: ACC, accuracy; R‐LRP, response‐locked LRP latency; RT, mean reaction time; Sex (F), female relative to male; S‐LRP, stimulus‐locked LRP latency.

^a^ Correlation between S‐LRP and R‐LRP latencies was marginally significant (*p* = 0.053).

### 2.3 Commonality Analysis Between S‐LRP and R‐LRP Latencies

To further justify dual mediation for predicting RT from age through LRP latencies (i.e., separate paths for S‐LRP and R‐LRP latencies), we conducted a set of commonality regression models to compare how much variance in RT and age is accounted for by shared variance between S‐LRP latency and R‐LRP latency and how much variance is accounted for by each latency measure independently. Importantly, these models were not interpreted causally; positioning age as the “outcome” was done solely to characterize the structure of shared and unique variance among the LRP latencies as they related to age. Results of this analysis are presented in Table 3.

**TABLE 3:** Commonality regression results for age and RT models.

	Age		RT	
	Proportion variance	% Total	Proportion variance	% Total
S‐LRP	0.0468	86.15	0.1834	85.58
R‐LRP	0.0031	5.73	0.0546	25.47
S‐LRP × R‐LRP	0.0044	8.11	−0.0237	−11.05
Total	0.0544	100.00	0.2143	100.00

*Note:* Coefficient represents the absolute proportion of the variance, while % total represents the proportion of the total variance accounted for by the model that is explained by that predictor. Negative variance is a common artifact of commonality analyses with predictors sharing little or no variance (see McMurray et al. 2025).

In the age model S‐LRP and R‐LRP latency together accounted for 5.4% of the variance in age (*R* ^2^ = 0.0544). S‐LRP latency accounted for 4.7% (86% of the explained variance), R‐LRP latency accounted for 0.3% (6%), while the shared variance between both accounted for 0.4% (8%), demonstrating relatively little overlap in age‐related variance between S‐LRP and R‐LRP latencies and supporting the rationale for separate mediation paths.

In the RT model, S‐LRP latency and R‐LRP latency together accounted for 21% of the variance in RT (*R* ^2^ = 0.2143). S‐LRP latency accounted for 18.3% (86% of the explained variance) and R‐LRP latency accounted for 5.5% (25%), while the shared variance between both latencies accounted for −2.4% (−11%). In commonality regressions, negative commonality coefficients are a common artifact produced by predictor variables with little to no shared variance (McMurray et al. 2025), suggesting that S‐LRP and R‐LRP latency contribute near‐completely independent variance in RT, supporting distinct mediation paths.

### 2.4 Primary SEM Mediation Results

We fit a SEM to test whether age predicted RT indirectly through distinct influences on S‐ and R‐LRP latencies. We predicted that age would predict RT (H1), that both S‐LRP and R‐LRP latencies would independently relate to RT (H2), and that age would indirectly predict RT through S‐LRP latency, R‐LRP latency, or both (H3).

**FIGURE 5:**
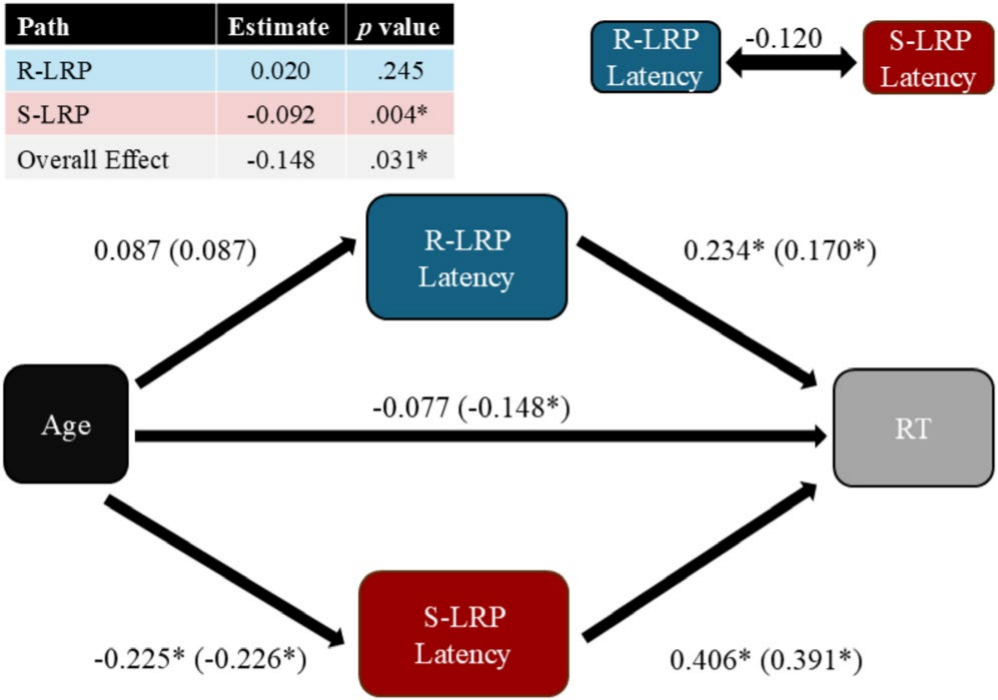
Visualization of the SEM testing mediation of age improvements in RT by S‐ and R‐LRP latencies. Strengths of indirect effects are noted in the table at top left, along with the total effect of age on RT. All regression weights are standardized. Covariance specified between the two mediators is indicated on the top right. Estimates on the arrows note estimates for each step in the model, while estimates in the parentheses compare the strength of the same effect in isolated bivariate linear models. Sex (not depicted) was also included in the model as an independent covariate regressing on RT (β = 0.221, *z* = 3.589, *p* < 0.001). *p < 0.05.

The model showed a significant overall effect of age on RT (*β* = −0.15, *z* = −2.16, *p* = 0.031), supporting H1, and a main effect of sex (*β* = 0.48, *z* = 3.59, *p* < 0.001). Both S‐LRP and R‐LRP latencies independently predicted RT (β_S‐LRP_ = 0.41, *z* = 6.39, *p* < 0.001; β_R‐LRP_ = 0.23, *z* = 3.77, *p* < 0.001), supporting H2. The model revealed a significant indirect effect of age on RT through S‐LRP latency (*ab* = −0.09, *z* = −2.88, *p* = 0.004), but not through R‐LRP latency (*ab* = 0.02, *z* = 1.16, *p* = 0.245). There was no direct effect of age on RT (*β* = −0.08, *z* = −1.22, *p* = 0.221), confirming that the negative relationship between age and RT was mediated by decreases in S‐LRP latency (and supporting H3). The model also included a residual covariance between S‐LRP and R‐LRP latencies, which was marginally significant (*β* = −0.12, z = −1.71, *p* = 0.087). The model is visualized in Figure 5.

## 3 Discussion

The present study investigated whether premotor and motor processing speeds relate differentially to age across adolescence, and whether they contribute differentially to age‐related improvements in overall processing speed. Consistent with prior studies (see Bucsuházy and Semela 2017; Vamsi et al. 2023; Kail 1991b), RT decreased in a generally linear fashion across age groups, with emerging‐adult participants performing the fastest (despite also being more accurate; see Figure 3). To further characterize this effect, we fractionated RTs using the LRP, based on the idea that S‐LRP latency corresponds with premotor processing speed (Gratton et al. 1988; Masaki et al. 2004) and R‐LRP latency corresponds with motor processing speed (Osman and Moore 1993; Osman et al. 1995). Consistent with the functional independence of processes reflected in the S‐LRP and R‐LRP (Masaki et al. 2004; Osman et al. 1995; Xu et al. 2015), S‐ and R‐LRP latencies in the current study were only modestly correlated and independently predicted RT. Importantly, our commonality analysis showed that S‐LRP and R‐LRP latencies accounted for almost no shared variance in RT, supporting the notion that each LRP interval reflects a distinct portion of the total response time, rather than a single, common speed factor. The common and unique variance found in both LRPs is consistent with cascade models of information processing, which argue that individual processes comprising RT are distinct but related (Coles et al. 1985).

### 3.1 Distinct Age‐Related Effects on Premotor and Motor Speeds

Consistent with the findings of Ridderinkhof and Van Der Molen (1997), S‐LRP latency improved with age, and furthermore, this relationship fully mediated age improvements in overall RT, even when controlling for R‐LRP latency. Importantly, this full mediation pattern does not suggest that all age‐related RT differences were accounted for by a single process, as the S‐ and R‐LRP index composites of multiple premotor and motor processes, respectively. Rather, the one‐sided mediation suggests that age differences in RT were influenced by the composite of premotor, but not motor, processes. Because the model was conducted on cross‐sectional data, these results should be interpreted descriptively rather than causally, as cross‐sectional mediation models often yield biased estimates relative to longitudinal designs (Maxwell and Cole 2007; Maxwell et al. 2011). Monte Carlo simulation indicated that the present sample was well‐powered to detect the indirect effect through S‐LRP (87.4%), but had limited sensitivity to detect an indirect effect through R‐LRP (13.0%), likely attributable to the small estimated effect size. Accordingly, the absence of a significant indirect path through R‐LRP should be interpreted cautiously, as it may reflect either a negligible effect or the sample's limited sensitivity to small effects in dual mediation.

R‐LRP latency did not relate to age in a zero‐order correlation nor in the full model, suggesting little‐to‐no maturation of motor processing during this window of adolescence (ages 14–19). Additionally, commonality regression showed very little unique effects of the R‐LRP and only minimal shared variance between the R‐LRP and S‐LRP relating to age, with most of the relationship between age and S‐LRP being accounted for by S‐LRP‐specific variance. This finding might appear inconsistent with the results of Śmigasiewicz et al. (2021), who reported that both premotor and motor speeds decrease at the same rate during childhood. However, conceptual and methodological differences between their study and the present one likely account for this discrepancy. First, as previously stated, using LRP onset instead of proximal EMG onset to fractionate RT divides premotor and motor intervals prior to central conduction, when the two domains are arguably more distinguishable. Second, whereas the present study focused on adolescence (ages 14–19), Śmigasiewicz and colleagues studied younger children (ages 6–14) in comparison to young adults, using the exponential model proposed by Kail (1991b) to estimate the developmental curve from childhood to maturity. These nonoverlapping age groups make direct comparison of the findings across the two studies challenging but suggest that any change in motor speed across the adolescent period is too small to detect in absence of a comparison to the earlier developmental period. Importantly, this is not the first report in the literature of motor speed showing little to no age differences across adolescence. In a well powered study (*N* = 593) using a variety of motor tasks varying in both complexity of movement and perceptual demands, Gasser et al. (2010) estimated that 70%–90% of all motor speed improvements achieved by adulthood occur prior to age 10, with the remainder by age 15–16. Taking this into consideration, it seems plausible that the simple motor processes involved in the flanker task mature prior to age 14, whereas other premotor processes continue to develop throughout adolescence.

Our results also conflict with Śmigasiewicz et al. (2021) interpretation of global maturation, which posits that faster, earlier‐developed motor processing systems slow down to match slower‐developing premotor processes to ensure a consistent flow of information. If this were the case, we should see a positive correlation between motor and premotor speeds. Instead, the present data showed the opposite, in that S‐LRP and R‐LRP latencies negatively correlated (though only marginally so). Rather than *slowing down* more fully developed processes to match slower ones, the results of the current study suggest that the system *speeds up* mature processes to compensate for lagging ones. To fully characterize developmental changes in premotor and motor processing speeds, future work could attempt to settle these discrepancies by combining approaches, recording EEG and distal EMG simultaneously in a sample spanning childhood and adolescence (e.g., ages 6–19).

### 3.2 Premotor and Motor Processing Speed Contributions to RT

The present study provided a precedent for investigating asynchronous functional development between distinct domains of processing speed. Age‐related differences in overall processing speed among adolescents were entirely explained by differences in premotor processing speed, with motor speed accounting for additional—but not age‐related—variance in individuals' overall response speed. In the full model, the regression coefficient for R‐LRP latency predicting RT exceeded its bivariate association, consistent with a suppression effect. This suggests that some shared variance with age and premotor speed—unrelated to overall processing speed—may obscure the motor speed contribution to total speed in simple correlations, while a multivariate model isolates the overall speed variance specifically attributed to motor processes. Nevertheless, this functional finding that age‐related difference in processing speed among adolescents is attributed to premotor—and not motor—speed aligns with structural neuroimaging evidence showing that motor areas reach physical maturity earlier than premotor areas (Giorgio et al. 2010; Lebel et al. 2008; O'Muircheartaigh et al. 2014; Tamnes et al. 2010). Lower‐level structural mechanisms may explain this asynchrony. Research has linked developmental change in processing speed to both axon myelination (Chevalier et al. 2015; Deoni et al. 2016; Ferrer et al. 2013; Scholz et al. 2009; Takeuchi et al. 2010; Zatorre et al. 2012) and synaptic pruning (Averbeck 2022; Liuzzi et al. 2023), both of which occur earlier in motor areas relative to premotor areas. Thus, it is possible that the distinct patterns of age‐related differences in the speeds of premotor and motor processes observed in the present study reflect differences in the timing of myelination and pruning in their respective cortical regions. While these structural findings provide a neurobiological context for the observed patterns, future longitudinal work integrating structural and functional measures will be critical to clarify how neuroanatomical differences translate into processing speed and to better bridge structural and functional accounts of brain development.

Differences in experience accrued with age may also contribute to the distinct patterns of age‐related differences in premotor and motor processing speeds. Across adolescence, individuals gain substantial experience with premotor processes like stimulus discrimination, rule‐based response selection, and inhibition of competing responses, which may contribute to differences in overall response speed (Goldstone 1998). It is possible that age‐related differences in premotor experiences are more pronounced in adolescence, while motor experiences show minimal variation. Longitudinal training‐based studies may help disentangle maturational and experiential contributions to development of domain‐specific processing speed.

### 3.3 Limitations

The present study's contributions must be viewed in the context of its limitations. Primarily, our sample represented only the period of adolescent development, and age differences were characterized between rather than within participants. A fuller understanding of domain‐specific influences on processing speed during development will require examination earlier in childhood and a longitudinal design in which the same individuals are assessed repeatedly as they age. Only this type of longitudinal replication would be able to establish whether differing age‐related effects on premotor and motor processing speeds truly stem from a developmental process. If employed with a sample beginning earlier in childhood, such an approach could allow researchers to estimate the “inflection point” at which domain trajectories diverge and, potentially, whether the point of such divergence varies across individuals as a function of various characteristics (e.g., intelligence, presence of developmental disorders). In addition, a longitudinal design would address the limits of cross‐sectional mediation models (Maxwell and Cole 2007; Maxwell et al. 2011), employing temporal precedence to make inferences about causal developmental processes. Nevertheless, the present findings suggest a precedent for utilizing LRP onset latency for further investigations of processing speed development.

In addition, the present sample had an uneven sex distribution (140 F, 64 M), leading to inflation of the standard error for sex differences, thereby potentially obscuring any such differences. Males had faster RTs than females, which is inconsistent with a large literature documenting females' faster performances across development (Burns and Nettelbeck 2005; Camarata and Woodcock 2006; Keith et al. 2011). However, sex differences were not observed in either S‐ or R‐LRP latency, nor did sex interact with age in predicting RT. Structurally, female brains age at a faster rate than male brains (Brouwer et al. 2021), and thus sex differences cannot be ruled out when discussing speed maturation. Additional work must be done to elucidate the role of sex in the development of domain‐specific processing speed.

## 4 Conclusion

By fractionating RTs using the LRP, we found that age‐related RT improvements in adolescent RT were mediated by faster premotor—and not motor—processing, providing evidence that motor processing speed may reach maturity earlier than premotor speed. Viewed through the lens of information‐processing accounts of development, the present findings are less consistent with the idea that processing speed maturation reflects only the influence of a single, global neurobehavioral processing factor (Kail 1991a) and more consistent with domain‐specific accounts in which distinct processes may mature asynchronously. Although our between‐subjects design cannot model developmental change directly, these findings provide a precedent for developmental researchers to investigate whether different neural processes follow asynchronous developmental timelines. Such findings would nuance the prevailing paradigm of a shared global processing speed factor. Furthermore, the present study demonstrates the utility of S‐ and R‐LRP latencies as functional measures of domain‐specific processing speed. This technique has been seldom used in the literature and could provide a valuable complement to structural imaging findings which have shown similarly asynchronous development of associated cortical regions. Altogether, the present study highlights how the LRP can link functional and structural accounts of development, clarifying how processing speed differs by age across domains.

## Author Contributions

**Bob McMurray:** conceptualization, writing – review and editing, validation, supervision, methodology. **Greg Hajcak:** writing – review and editing, conceptualization, funding acquisition, validation, supervision. **William Slawson:** conceptualization, investigation, writing – original draft, writing – review and editing, formal analysis, methodology, visualization, data curation, software. **Bruce D. Bartholow:** conceptualization, funding acquisition, writing – review and editing, validation, project administration, supervision, writing – original draft.

## Funding

This work was supported by grant R01AA030278 from the National Institute on Alcohol Abuse and Alcoholism (Bartholow, PI).

## Conflicts of Interest

The authors declare no conflicts of interest.

## Supporting information


**Data S1:** psyp70313‐sup‐0001‐Supinfo.docx.

**Data S2:** psyp70313‐sup‐0002‐Supinfo1.docx.

## Data Availability

The data that support the findings of this study are available from the corresponding author upon reasonable request.

## Endnotes

^1^
Exponential iterations of each model are presented in the Supporting Information.
