# Supplementary material for: Age‐Related Differences in Response Time Across Adolescence Reflect Premotor, but Not Motor, Processing Speed
Source: Psychophysiology. 2026 May 6;63(5):e70313. doi: 10.1111/psyp.70313 (PMC13150053; doi:10.1111/psyp.70313)
Supplement: Supplementary file 1 — Data S1: psyp70313‐sup‐0001‐Supinfo.docx. [file PSYP-63-e70313-s002.docx]

**Supplementary Materials:**

***Improvements in Response Latency During Adolescence Reflect More Efficient Premotor Selection, Not Enhanced Motor Speed***

W. Slawson, G. Hajcak, B. McMurray, & B. D. Bartholow

**Exponential Age x Processing Speed Model**

To be more consistent with extant literature on the development of processing speed (see Kail 1991b), we also ran our primary model with an exponential design by substituting the natural log of age for age in the SEM. This altered model produced results consistent with those of the linear model. Age showed a significant indirect effect on RT through S-LRP latency (*ab* = -0.090, *z* = -2.829, *p* = .005), but not through R-LRP latency (*ab* = 0.021, *z* = 1.183, *p* = .237). The main effect of age diminished (β = -0.077, *z* = -1.220, *p* = .222), and the overall effect was significant (β = -0.146, *z* = 2.117, *p* = .034).

**Tests for Invariance Across Flanker Compatibility**

To examine whether the results of the primary study SEM varied across compatible and incompatible trials, we used a multi-group SEM to test for invariance We first ran an unconstrained model in which regression paths were estimated freely for each trial type, and then a constrained model in which all regression paths were forced to be equal across trial types, finally comparing the two models with a chi-square difference test to evaluate invariance. The test supported invariance (Δχ² (8) = 13.064, *p* = 0.109), suggesting the SEM did not fit differently across trial types.

**Test of Interaction between LRP Latencies and Sex**

To test for two- and three-way interactions between LRP latencies and sex when predicting RT, we ran an exploratory ordinary least squares multiple regression with RT as the outcome, and S-LRP latency, R-LRP latency, and sex as predictors with interactions specified. S-LRP and R-LRP latencies were mean-centered to improve interpretability of lower order terms in the presence of interactions, and to reduce nonessential multicollinearity between lower order terms and their interactions with sex (Schielzeth, 2010).

The overall multiple regression model was significant, *F*(7,186) = 10.80, p < .001, explaining 28.9% of the variance in RT (R^2^ = .289, adjusted R^2^ = .262; N = 194). None of the specified interactions were significant: S-LRP x R-LRP latency (β = -0.104, *t* = -1.357, *p* = .176), S-LRP x Sex (β = 0.104, *t* = 0.663, *p* = .508), R-LRP x Sex (β = 0.150, *t* = 0.935, *p* = .351), nor the S-LRP x R-LRP x Sex interaction (β = -0.219, *t* = -1.099, *p* = .273). Thus, there was no evidence that the relationships between LRP latencies and RT varied with one another, nor with sex.

**Exploratory Analysis of Age Differences in LRP Amplitude**

LRP amplitudes were calculated by averaging voltages during a 100-ms window of activity around the peak positive amplitude within 200-500 ms after stimulus onset (S-LRP) and -200-0 ms prior to response execution (R-LRP).

To examine age-related differences in LRP amplitude, we fit a regression model in which age predicted LRP amplitude, with LRP latency as a covariate to control for the latency effects reported in the main text. We also ran additional models regressing RT and accuracy onto LRP amplitude to assess whether LRP amplitude predicted task outcomes, with LRP latency again added as a covariate. We ran separate models for S- and R-LRP amplitudes to account for the theoretical overlap between the two. Sex was included as a covariate in all models.

Age positively predicted LRP amplitude (Figure 2) in both the stimulus-locked (β = 0.231, *t* = 3.376, *p* < .001) and response-locked alignments (β = 0.210, *t* = 3.251, *p* = .001). Independently of age, LRP amplitude also predicted LRP latency in both the stimulus-locked (β = -0.292, *t* = -4.112, *p* < .001) and response-locked ERPs (β = 0.413, *t* = 6.047, *p* < .001) in an opposing manner: larger LRP amplitudes were associated with shorter S-LRP latencies and longer R-LRP latencies.

LRP amplitude also predicted accuracy in both the stimulus-locked alignment (β = 0.161, *t* = 2.151, *p* = .033) and marginally in the response-locked alignment (β = 0.128, *t* = 1.659, *p* = .099), such that larger LRP amplitudes were associated with better accuracy. Neither S-LRP amplitude (β = 0.000, *t* = 0.004, *p* = 0.996) nor R-LRP amplitude (β = -0.089, *t* = -1.180, *p* = 0.240) predicted RT.

While exploratory in nature (hence requiring cautious interpretation), analysis of LRP amplitude suggests synaptic pruning as a candidate mechanism for age-related improvements in processing speed. The positive association between LRP amplitude and accuracy observed here replicates the result reported by Wild-Wall et al. (2008), wherein older participants had larger LRP amplitudes and were more accurate in a flanker task than were young adults. Wild-Wall and colleagues suggested that amplitude increases were the result of age-related slowing of flanker (relative to target) interference from visual to motor areas—with slower transmission allowing for more precise response selection.

The fact that we observed the same pattern in adolescents requires an alternative explanation in that participants whose LRP amplitudes were larger (i.e., older vs. younger adolescents) were *faster* in the task. We speculate that synaptic pruning in adolescence reduces transient conduction of the response selection signal to the contralateral motor cortex (see Liuzzi et al., 2023). Having fewer redundant synapses reduces ‘noise’ in the hemisphere of motor cortex mapped to *incorrect* responses, producing a larger difference wave in the LRP. Increased LRP amplitudes, then, may reflect a more specified neural path for the response selection signal carved by synaptic pruning. Testing this hypothesis would require direct examination with participants who represent a wider range of development (e.g., pre-teen to older adult).
