# Supplementary material for: Age‐Related Differences in Response Time Across Adolescence Reflect Premotor, but Not Motor, Processing Speed
Source: Psychophysiology. 2026 May 6;63(5):e70313. doi: 10.1111/psyp.70313 (PMC13150053; doi:10.1111/psyp.70313)
Supplement: Supplementary file 2 — Data S2: psyp70313‐sup‐0002‐Supinfo1.docx. [file PSYP-63-e70313-s001.docx]

**Supplementary Materials:**

***Improvements in Response Latency During Adolescence Reflect More Efficient Premotor Selection, Not Enhanced Motor Speed***

W. Slawson, G. Hajcak, B. McMurray, & B. D. Bartholow

**Exponential Age x Processing Speed Models**

To be more consistent with extant literature on the development of processing speed (see Kail 1991b), we also ran our age-speed models (hypotheses 1 and 3) with an exponential design by substituting the natural log of age for age in all models. Results for these exponential models were consistent with the linear models. Age predicted RT (β = -5.967, *t* = -2.063, *p* = 0.041), showing no significant interaction with sex (β = -5.852, *t* = -0.912, *p* = .363) or flanker compatibility (β = -0.600, *t* = -0.569, *p* = .570), and no significant three-way interaction (Age x Sex x Compatibility; β = 2.221, *t* = 0.945, *p* = .364). The log of age correlated with S-LRP latency (*r* = -.22, *p* = .002), but not with R-LRP latency (*r* = .09, *p* = .221).

Entering the natural log of age into the SEM also produced results consistent with those of the linear model. Age showed a significant indirect effect on RT through S-LRP latency (*ab* = -0.090, *z* = -2.829, *p* = .005), but not through R-LRP latency (*ab* = 0.021, *z* = 1.183, *p* = .237). The main effect of age diminished (β = -0.077, *z* = -1.220, *p* = .222), and the overall effect was significant (β = -0.146, *z* = 2.117, *p* = .034).

**Tests for Invariance Across Flanker Compatibility**

To examine whether the results of hypotheses 2 and 3 varied across compatible and incompatible trials, we used a multi-group SEM to test for invariance We first ran an unconstrained model in which regression paths were estimated freely for each trial type, and then a constrained model in which all regression paths were forced to be equal across trial types, finally comparing the two models with a chi-square difference test to evaluate invariance.

The model regressing RT onto S- and R-LRP latencies (hypothesis 2) showed marginally significant variance (Δχ² (8) = 7.280, *p* = 0.063). The unconstrained model showed that the slope of S-LRP latency on RT was much greater for incompatible trials (β = 0.496, *z* = 6.142, *p* = .000) relative to compatible ones (β = 0.273, *z* = 5.048, *p* = .000). This finding replicates previous research (Gratton et al., 1988; Kappenman et al., 2012) and aligns with the accepted interpretation that the flanker effect reflects a delay resulting from increased conflict in stimulus evaluation and response selection processes. Thus, it stands to reason that we would see a greater relationship between S-LRP and RT on incompatible trials (as response conflict increases variance in premotor processing speed). However, there was also a moderate difference in the slope of the R-LRP latency on RT between incongruent (β = 0.273, *z* = 4.011, *p* = .000) and congruent trials (β = 0.113, *z* = 1.762, *p* = .078), providing weak evidence that the flanker effect impacts motor execution processes in addition to premotor ones. This effect has been observed in the literature as well (see Nikolaev et al., 2008), aligning with continuous flow models of processing in which effects on one process can leak onto others within the cascade (Coles et al., 1985). However, because the overall S-LRP x R-LRP interaction effect in the present study was not significant, this finding should be interpreted with caution.

The mediation model for hypothesis 3 supported invariance (Δχ² (8) = 13.064, *p* = 0.109), suggesting the model did not fit differently across trial types.

**Exploratory Analysis of Age Differences in LRP Amplitude**

LRP amplitudes were calculated by averaging voltages during a 100-ms window of activity around the peak positive amplitude within 200-500 ms after stimulus onset (S-LRP) and -200-0 ms prior to response execution (R-LRP).

To examine age-related differences in LRP amplitude, we fit a regression model in which age predicted LRP amplitude, with LRP latency as a covariate to control for the latency effects reported in the main text. We also ran additional models regressing RT and accuracy onto LRP amplitude to assess whether LRP amplitude predicted task outcomes, with LRP latency again added as a covariate. We ran separate models for S- and R-LRP amplitudes to account for the theoretical overlap between the two. Sex was included as a covariate in all models.

Age positively predicted LRP amplitude (Figure 2) in both the stimulus-locked (β = 0.231, *t* = 3.376, *p* < .001) and response-locked alignments (β = 0.210, *t* = 3.251, *p* = .001). Independently of age, LRP amplitude also predicted LRP latency in both the stimulus-locked (β = -0.292, *t* = -4.112, *p* < .001) and response-locked ERPs (β = 0.413, *t* = 6.047, *p* < .001) in an opposing manner: larger LRP amplitudes were associated with shorter S-LRP latencies and longer R-LRP latencies.

LRP amplitude also predicted accuracy in both the stimulus-locked alignment (β = 0.161, *t* = 2.151, *p* = .033) and marginally in the response-locked alignment (β = 0.128, *t* = 1.659, *p* = .099), such that larger LRP amplitudes were associated with better accuracy. Neither S-LRP amplitude (β = 0.000, *t* = 0.004, *p* = 0.996) nor R-LRP amplitude (β = -0.089, *t* = -1.180, *p* = 0.240) predicted RT.

While exploratory in nature (hence requiring cautious interpretation), analysis of LRP amplitude suggests synaptic pruning as a candidate mechanism for age-related improvements in processing speed. The positive association between LRP amplitude and accuracy observed here replicates the result reported by Wild-Wall et al. (2008), wherein older participants had larger LRP amplitudes and were more accurate in a flanker task than were young adults. Wild-Wall and colleagues suggested that amplitude increases were the result of age-related slowing of flanker (relative to target) interference from visual to motor areas—with slower transmission allowing for more precise response selection.

The fact that we observed the same pattern in adolescents requires an alternative explanation in that participants whose LRP amplitudes were larger (i.e., older vs. younger adolescents) were *faster* in the task. We speculate that synaptic pruning in adolescence reduces transient conduction of the response selection signal to the contralateral motor cortex (see Liuzzi et al., 2023). Having fewer redundant synapses reduces ‘noise’ in the hemisphere of motor cortex mapped to *incorrect* responses, producing a larger difference wave in the LRP. Increased LRP amplitudes, then, may reflect a more specified neural path for the response selection signal carved by synaptic pruning. Testing this hypothesis would require direct examination with participants who represent a wider range of development (e.g., pre-teen to older adult).
